# Trait correlated expression combined with eQTL and ASE analyses identified novel candidate genes affecting intramuscular fat

**DOI:** 10.1186/s12864-021-08141-9

**Published:** 2021-11-08

**Authors:** Yan Liu, Huan Long, Simin Feng, Tingting Ma, Mufeng Wang, Lizhu Niu, Xinyi Zhang, Lianni Wang, Yu Lei, Yilong Chen, Qiankun Wang, Xuewen Xu

**Affiliations:** 1 Key Laboratory of Agricultural Animal Genetics, Breeding and Reproduction, Ministry of Education & College of Animal Science and Technology, Huazhong Agricultural University, Wuhan, 430070 China; 2 The Cooperative Innovation Center for Sustainable Pig Production, Wuhan, 430070 China; 3 Key Lab of Swine Genetics and Breeding of Ministry of Agriculture and Rural Affairs, Wuhan, 430070 China

**Keywords:** Pig, IMF, eQTL, ASE, GWAS, AMPK/PPAR

## Abstract

**Background:**

Intramuscular fat (IMF) content is a determining factor for meat taste. The Luchuan pig is a fat-type local breed in southern China that is famous for its desirable meat quality due to high IMF, however, the crossbred offspring of Luchuan sows and Duroc boars displayed within-population variation on meat quality, and the reason remains unknown.

**Results:**

In the present study, we identified 212 IMF-correlated genes (FDR ≤ 0.01) using correlation analysis between gene expression level and the value of IMF content. The IMF-correlated genes were significantly enriched in the processes of lipid metabolism and mitochondrial energy metabolism, as well as the AMPK/PPAR signaling pathway. From the IMF-correlated genes, we identified 99 genes associated with expression quantitative trait locus (eQTL) or allele-specific expression (ASE) signals, including 21 genes identified by both cis-eQTL and ASE analyses and 12 genes identified by trans-eQTL analysis. Genome-wide association study (GWAS) of IMF identified a significant QTL on SSC14 (*p*-value = 2.51*E*^−7^), and the nearest IMF-correlated gene *SFXN4* (*r* = 0.28, FDR = 4.00*E*^−4^) was proposed as the candidate gene. Furthermore, we highlighted another three novel IMF candidate genes, namely *AGT*, *EMG1*, and *PCTP*, by integrated analysis of GWAS, eQTL, and IMF-gene correlation analysis.

**Conclusions:**

The AMPK/PPAR signaling pathway together with the processes of lipid and mitochondrial energy metabolism plays a vital role in regulating porcine IMF content. Trait correlated expression combined with eQTL and ASE analysis highlighted a priority list of genes, which compensated for the shortcoming of GWAS, thereby accelerating the mining of causal genes of IMF.

**Supplementary Information:**

The online version contains supplementary material available at 10.1186/s12864-021-08141-9.

## Background

Intramuscular fat (IMF) is the adipose tissue deposited between skeletal muscle fibers, and its content is positively correlated with meat juiciness, flavor intensity, and tenderness [1]. A higher IMF content (> 3.5%) of pork is generally accepted because of its positive sensory experiences. However, too much visible fat in the meat will reduce its consumer acceptability due to health concerns [1]. An optimal range of IMF content of 2.0 to 4.0% was recommended as the industry target in the United States according to previous studies [2], and the IMF content has moderate to high heritability: the average heritability is 0.4, ranging from 0.24 to 0.67 in different populations [3–5]. Despite its high heritability, direct selection for IMF content during pig breeding is difficult to put into practice since it is measured post-slaughter. Therefore, seeking the regulatory genes and potential molecular markers of IMF content is an important task for genetic research and pig breeding.

To specifically reveal the genes and pathways involved in intramuscular fat deposition, muscle transcriptomes based on cDNA microarray were compared between extreme animals with the divergent selection of IMF content, such as 2 × 8 Pietrain x Duroc F2 animals [6], 2 × 35 Duroc pigs [7] and 2 × 20 Iberian (25%) x Landrace (75%) back-crossed animals [8]. Recently, the RNA sequencing (RNA-seq) approach was used to investigate muscle transcriptome differences in Duroc pigs with distinct lipid profiles [9] and Berkshire pigs with divergent IMF content [10], which highlighted the vital effect of lipid metabolism-related genes, such as fatty-acid synthase (*FAS*N) and stearoyl-CoA desaturase (*SCD*), in IMF content determination. Although the comparative transcriptome profiling approach identified some differential expressed genes (DEGs) related to IMF content, it was hard to prioritize candidate genes that genetically contributed to phenotypic variation. Instead, the approach combining high-throughput genotyping and gene expression analysis, namely expression quantitative trait loci (eQTL) and allele specific expression (ASE) analyses, could discover potential genomic variants that exerted their effects on phenotypic variation by regulating gene expression level. Thus, eQTL and ASE analyses were often used to prioritize causal genes in the genome-wide association studies (GWASs) of complex traits, such as porcine muscle glycogen content [11] and intramuscular fat [12].

The Luchuan pig is a typical Chinese local variety with high fat deposition and good meat quality, widely distributed in southern China, including Guangdong and Guangxi provinces. In recent years, Luchuan sows were crossed with Duroc boars to produce high-quality meat, whereas large within-population variations of fat deposition and meat quality were observed. To identify potential genes controlling meat quality traits in this population, we collected skeletal muscle samples for trait measurement and RNA-seq. Recently, we reported the results of genome-wide eQTL and ASE analyses based on RNA-seq data, in which we confirmed some known candidate genes and revealed some novel candidate genes for meat quality traits [13]. In the present study, we performed the correlation analysis between gene expression level and IMF content, and GWAS of IMF. Further, we conducted an integrated analysis of IMF-correlated genes, eQTL, and GWAS, through which we hope to reveal important pathways and candidate genes affecting IMF content.

## Results

### Correlation analysis with trait and gene expression levels identified 212 IMF-correlated genes

**Fig. 1:**
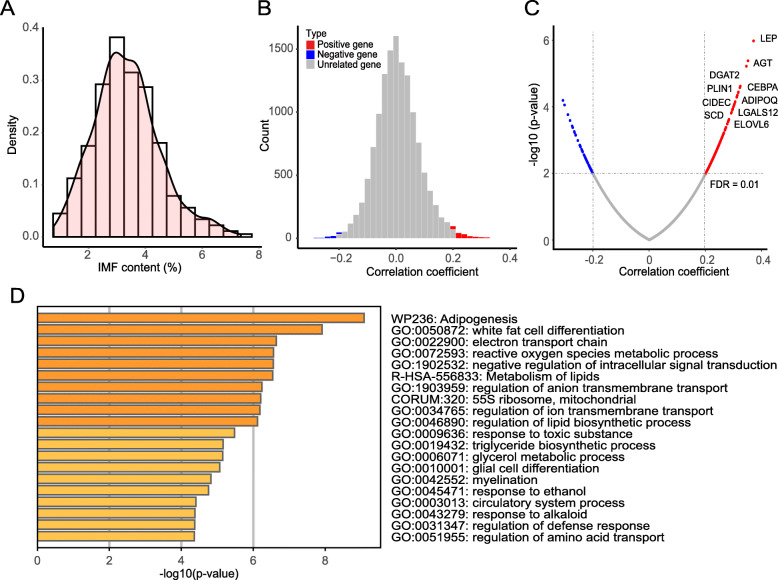
Characterization of IMF-correlated gene. (**A**). The density distribution of IMF content of 425 individuals. (**B**). Histogram of the Pearson correlation coefficients of IMF and gene expression level. The red represents the positively correlated genes and the blue represents the negatively correlated genes. (**C**). Scatter plot represents the distribution of correlation coefficients (X-axis) and – log_10_ (*p*-value) (Y-axis). The red and blue colors represent positively and negatively correlated genes respectively. The gray dashed lines indicate the selected cut-off of FDR (FDR = 0.01) and the corresponding correlation coefficient. (**D**). Top 20 enrichment clusters of 212 IMF-correlated genes

As described in our recent paper, the F1 animals of Duroc boars and Luchuan sows displayed great phenotypic variation on meat quality [13]. For IMF content of longissimus dorsi muscle, the 425 F1 animals had an average IMF of 3.35% ± 1.16%, ranging from 0.77 to 7.36% (Fig. 1A). We randomly selected 189 individuals from the population for RNA sequencing with longissimus dorsi muscle, by which the expression levels of all reference genes (25,880 genes) were estimated and a total of 13,450 genes were kept for eQTL and ASE analysis after the QC procedure [13]. Here, we conducted the correlation analysis between IMF and gene expression levels. To do this, the log_2_-transformed gene expression level and the phenotypic values of IMF were pre-adjusted for systematic effects using a fixed linear model. Within the 13,450 input genes, correlation analysis identified 212 genes that were significantly correlated with IMF (FDR ≤ 0.01) (Supplementary Table S1). The 212 genes contained 154 negatively correlated genes and 58 positively correlated genes, and the absolute value of the correlation coefficient ranged from 0.20 to 0.37 (Fig. 1B & 1C). These genes were significantly enriched in 20 clusters, which included the processes or GO terms related to adipogenesis, lipid biosynthetic and metabolism process, triglyceride biosynthetic process, electron transport chain, mitochondrial translation and so on (Fig. 1D, Supplementary Table S2).

### The IMF-correlated genes were significantly enriched in the processes of lipid and energy metabolism and the AMPK/PPAR signaling pathway

**Fig. 2:**
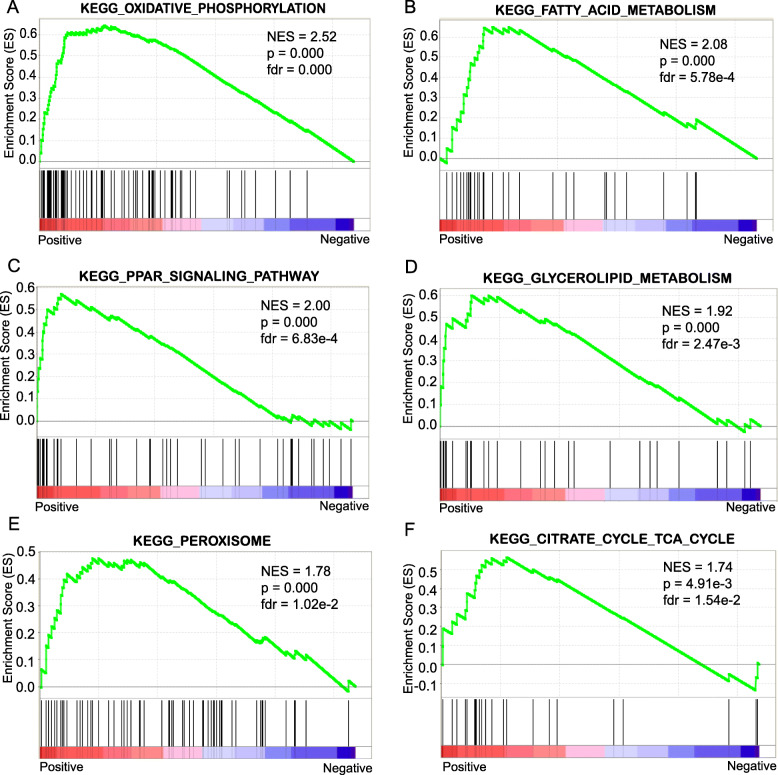
GSEA analysis for all IMF-correlated genes. Six significantly enriched KEGG pathways were output as follows: (**A**). Oxidative phosphorylation, (**B**). Fatty acid metabolism, (**C**). PPAR signaling pathway, (**D**). Glycolipid metabolism, (**E**). Peroxisome and (**F**). Tricarboxylic acid cycle. NES means normalized enrichment score. Permission has been obtained from Kanehisa laboratories to use the KEGG pathway database [48]

To explore potential signaling pathways affecting IMF, we carried out KEGG, GSEA, and protein-protein interaction (PPI) network analysis using the 212 IMF-correlated genes. The KEGG pathway analysis revealed that five IMF-correlated genes including *PCK1*, *PLIN1*, *PPARG*, *SCD*, and *ADIPOQ* were significantly enriched in the PPAR signaling pathway (q-value = 2.12*E*^−2^), six genes including *NDUFB8*, *NDUFS3*, *ATP6V1F*, *UQCRQ*, *UQCR10*, and *NDUFB11* were enriched in oxidative phosphorylation (q-value = 3.61*E*^−2^), and seven genes including *FASN*, *LEP*, *PCK1*, *PPARG*, *SCD*, *ADIPOQ*, and *STRADB* were enriched in the AMPK signaling pathway (q-value = 6.98*E*^−3^) (Supplementary Table S2). The GSEA analysis with the KEGG gene sets as background revealed that 22 enriched gene sets were positively related with IMF and 24 gene sets were negatively related with IMF (Supplementary Table S3). Oxidative phosphorylation was the most significantly enriched pathway with the highest positive enrichment score (NES = 2.52), followed by fatty acid metabolism (NES = 2.08), PPAR signaling pathway (NES = 2.00) and glycolipid metabolism (NES = 1.92), peroxisome (NES = 1.78) and tricarboxylic acid cycle (NES = 1.74) (Fig. 2).

**Fig. 3:**
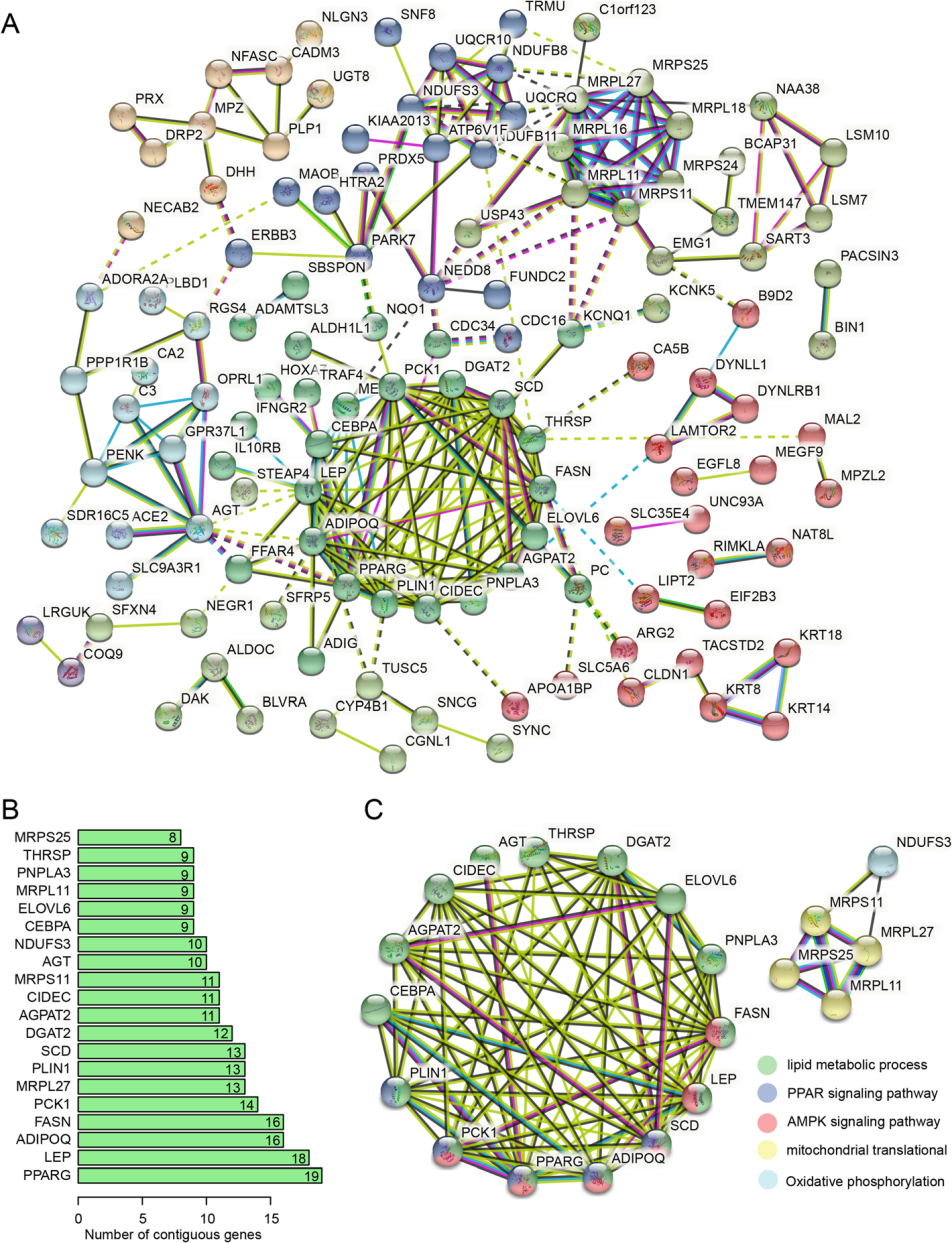
PPI network analysis of IMF-correlated genes. (**A**). PPI network of 124 IMF-correlated genes. Circles represent genes (nodes) and lines represent the interaction between genes (edges). Line colors represent the different extent of evidence of PPI and circle colors represent different clusters generated by k-means clustering. (**B**). Barplot of edges number of top 20 core genes in the PPI network. (**C**). PPI sub-network of top 20 core genes. Different circle colors represent distinct functional processes or pathways

Furthermore, we used the STRING database to explore the co-expression or interaction of 212 IMF-correlated genes. Among these genes, only 178 genes could be discerned by the STRING database and kept for PPI enrichment analysis. Finally, 124 nodes (genes) and 253 edges were shown in the network (Fig. 3A), whereas another 54 genes were hidden since they did not connect with any nodes. The network contained a significantly higher frequency of interactions than the expected by evaluating the expected edges for a random set of proteins of similar size (expected edges = 123, PPI enrichment *p*-value < 1.00*E*^−16^). In the PPI network of IMF-correlated genes, the genes related to lipid biosynthesis or metabolic processes displayed an extremely high frequency of interactions, which was followed by the genes related to mitochondrial translation and the oxidative phosphorylation pathway (Fig. 3A). We counted the degree of connectivity (edges) for each gene, and *PPARG* had the highest frequency of interactions (Fig. 3B). The top 20 core genes, such as *PPARG*, *LEP, ADIPOQ*, *FASN*, *PCK1* and so on, had the minimum edge of 8 (Fig. 3B), with which a sub-core network was built (Fig. 3C). Interestingly, the 20 core genes were mainly enriched in lipid metabolic process (*n* = 15), PPAR signaling pathway (*n* = 5), AMPK signaling pathway (*n* = 6) and mitochondrial translation (*n* = 4) (Fig. 3C).

### Intersection of IMF-correlated genes and target genes of cis-eQTL, ASE and trans-eQTL

**Fig. 4:**
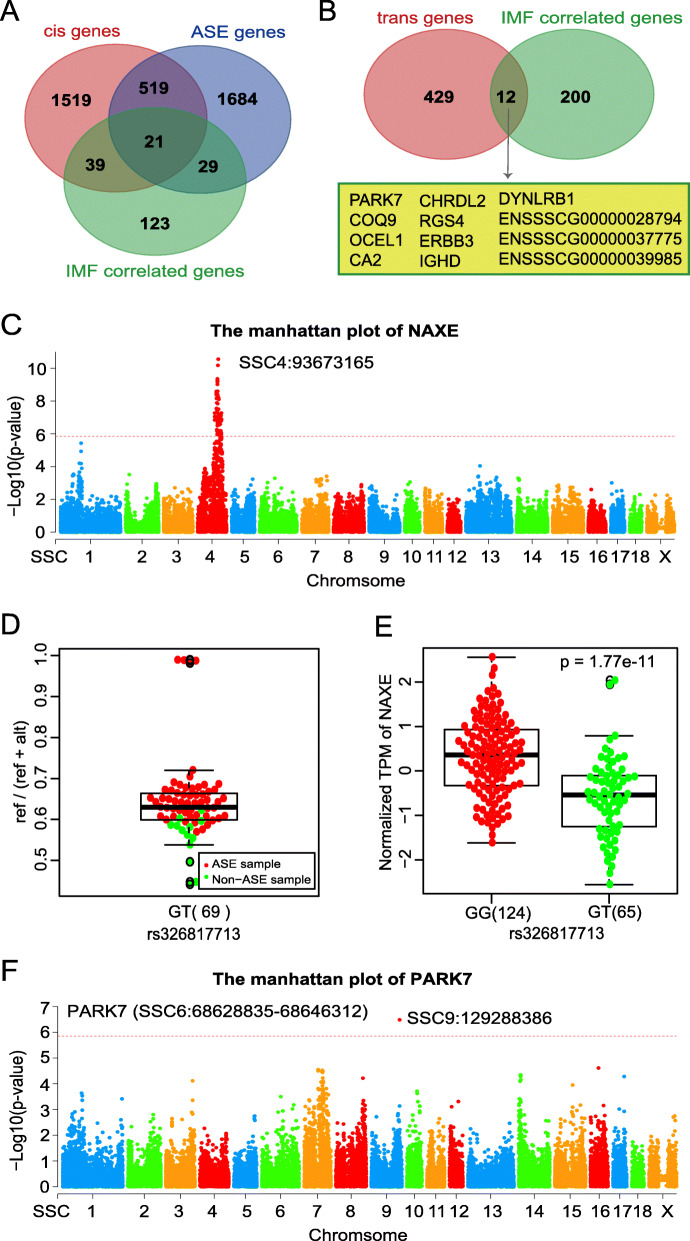
eQTL and ASE analyses of IMF-correlated genes. (**A**). Veen diagram of significantly cis-eQTL target genes, ASE genes and IMF-correlated genes. (**B**). Veen diagram of significantly specific trans-eQTL target genes and IMF-correlated genes. (**C**). The Manhattan plot of *NAXE*. The red dashed line represents the cutoff of statistical significance at the genome-wide level (*p* = 0.05/36045). (**D**). Boxplot of the allele-specific expression ratio of *NAXE* for the SNP of rs326817713 (G > T). Allele-specific expression ratio means the number of reads aligned to the reference allele divided by the total number of reads aligned to this site. “ASE sample” (red dot) indicates the heterozygotes with ASE signal at this site, and “Non-ASE sample” (green dot) indicates the heterozygotes without ASE signal at this site. (E). Boxplot of the expression level of *NAXE* with different genotypes of rs326817713. (**F**). The Manhattan plot of *PARK7*. The red dashed line represents the cutoff of statistical significance at the genome-wide level (*p* = 0.05/36,045)

Our previous study identified 2098 genes with cis-eQTL signals, 441 genes with specific trans-eQTL signals (without cis-eQTL signals) and 2253 genes with allele-specific expression (ASE) signals [13]. Among the 212 IMF-correlated genes, there were 60 cis-eQTL-associated genes and 50 ASE genes (Supplementary Table S1), in which 21 genes were identified by both cis-eQTL and ASE analyses (Table 1, Fig. 4A). In addition, the 212 IMF -correlated genes contained 12 trans-eQTL-associated genes (Fig. 4B, Supplementary Table S1). Taking the *NAXE* gene as an example, whose expression level were positively correlated with IMF (*r* = 0.24, FDR = 1.90*E*^−3^), and a strong cis-eQTL was identified at SSC4:93673165 (*p*-value = 2.76*E*^−11^) (Fig. 4C). ASE analysis revealed that the reference allele of the SNP (rs326817713) in the first exon of *NAXE* displayed a relatively high expression level for 56 individuals of 69 heterozygotes (Fig. 4D). Meanwhile, the polymorphism of rs326817713 was significantly associated with the expression level of *NAXE* (*p*-value = 1.77*E*^−11^) (Fig. 4E). Among the 12 trans-eQTL-associated genes (Supplementary Table S1), the expression of *PARK7* had the most significant correlation with IMF (*r* = 0.26, FDR = 9.00*E*^−4^). A significant trans-eQTL on chromosome 9 (rs325298336, *p*-value = 3.25*E*^−7^) was identified for *PARK7* that located on chromosome 6 (Fig. 4F).

**Table 1:** The overlap between eQTL analysis, ASE analysis and correlation analysis

gene_name	eQTL analysis			ASE analysis				Correlation analysis		
	SNP_id	***p***value	FDR	SNP_id	Het	ASE	ratio	cor	***p***value	FDR
EMG1	5:64350471	2.28E-29	1.02E-24	5:63751365	103	103	1.00	0.215	5.76E-03	6.20E-03
PCTP	12:32427760	2.80E-12	2.74E-09	12:32062640	43	11	0.26	0.212	6.43E-03	7.30E-03
NAXE	4:93673165	2.76E-11	2.03E-08	4:93495840	69	56	0.81	0.243	1.72E-03	1.90E-03
EIF2B3	6:167199552	2.00E-10	1.17E-07	6:166438671	49	10	0.20	−0.211	6.75E-03	7.10E-03
SLC9A3R1	12:6284901	1.04E-08	3.55E-06	12:6468404	160	98	0.61	0.208	7.47E-03	9.00E-03
ZFAND2B	15:120963216	1.48E-08	4.85E-06	15:121243305	74	73	0.99	0.242	1.78E-03	2.00E-03
MRPS11	1:191048963	1.49E-07	3.63E-05	1:191321445	132	61	0.46	0.242	1.82E-03	1.90E-03
C1orf123	6:158874827	6.78E-07	1.34E-04	6:159011592	43	21	0.49	0.206	8.11E-03	8.90E-03
PRDX5	2:7551456	1.01E-06	1.86E-04	2:7803041	88	10	0.11	0.245	1.60E-03	1.60E-03
MRPL16	2:11716316	1.01E-06	1.86E-04	2:11694100	62	29	0.47	0.209	7.25E-03	7.40E-03
MGST2	8:86943096	4.96E-06	6.83E-04	8:87328624	149	86	0.58	0.240	1.94E-03	1.90E-03
IFNGR2	13:196334437	6.58E-06	8.67E-04	13:197020918	170	16	0.09	0.197	1.15E-02	9.90E-03
IL10RB	13:196629902	1.00E-05	1.21E-03	13:196860215	39	19	0.49	0.202	9.40E-03	8.00E-03
DGAT2	9:9831161	1.02E-05	1.24E-03	9:10063315	152	117	0.77	0.322	2.68E-05	1.00E-05
SMIM20	8:19726922	2.81E-05	2.77E-03	8:19714368	175	143	0.82	0.269	4.93E-04	1.10E-03
AGT	14:59704514	3.81E-05	3.54E-03	14:59648664	177	85	0.48	0.345	6.09E-06	1.00E-05
ATP6V1F	18:20146282	5.54E-05	4.72E-03	18:19756485	166	76	0.46	0.316	3.78E-05	1.00E-04
PLBD1	5:57889765	7.38E-05	5.94E-03	5:57951366	82	16	0.20	−0.265	5.97E-04	3.00E-04
CDC34	2:77362555	9.82E-05	7.42E-03	2:77768209	188	96	0.51	0.249	1.32E-03	1.80E-03
FRMD3	10:31523619	2.91E-04	1.68E-02	10:31714269	115	25	0.22	0.228	3.32E-03	2.40E-03
ENSSSCG00000032003	2:5854819	5.83E-04	2.82E-02	2:4978950	183	135	0.74	0.251	1.18E-03	1.20E-03

“Het” represents the total number of observed heterozygotes, and “ASE” means the number of heterozygotes that displayed ASE. “cor” represents the Pearson correlation coefficient

### Integration of GWAS, eQTL analysis, and IMF-gene correlation analysis highlighted four novel candidate genes of IMF

**Fig. 5:**
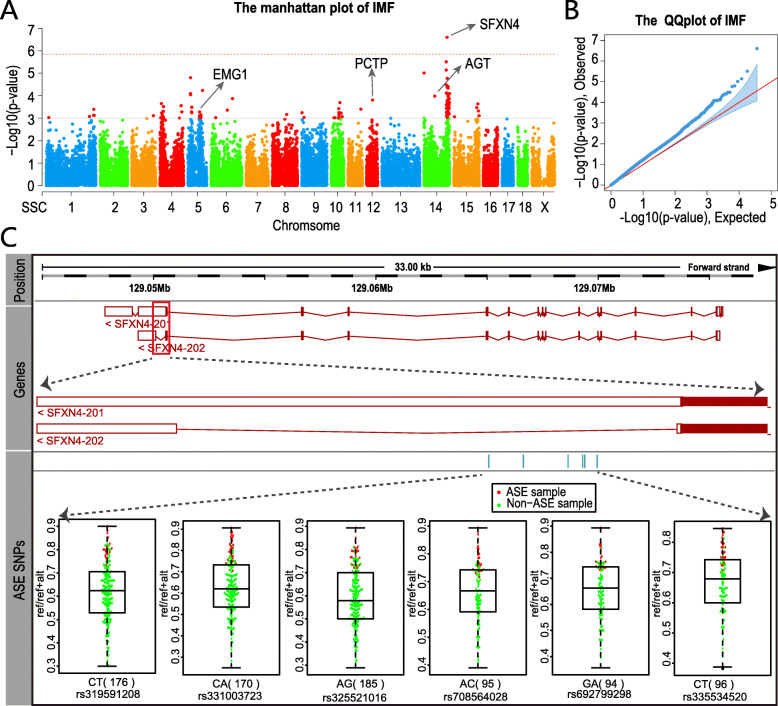
GWAS of IMF using the fixed linear model. (**A**). The Manhattan plot of IMF. The red dashed line represents the cutoff of statistical significance at the genome-wide level (*p* = 0.05/36,045) and the gray dotted line represents the cutoff of *p* = 1.00*E*^−3^. (**B**). The QQplot of IMF. (**C**). The genomic structure of *SFXN4* and its ASE signals. The first layer indicates the location of the genome. The middle layer indicates the gene structure of *SFXN4*, and the bottom layer indicates six ASE-SNPs and their allele-specific expression

Integration of eQTL (or ASE) analysis and trait correlated expression analysis established the relationship of SNP-gene-trait, whereas the association between SNP and trait remains unknown. To establish the potential triple relationship of SNP-gene-trait, we conducted genome-wide association study for IMF using a fixed linear model with the 189 animals that were genotyped using the Illumina porcine 50 K + SNP iSelect™ BeadChip [13]. It revealed that only one SNP on chromosome 14 (WU_10.2_14_139488835, *p*-value = 2.51*E*^−7^) was associated with IMF on the level of genome-wide significance (*p*-value = 1.39*E*^−6^) (Fig. 5A & 5B). From the list of IMF-correlated genes, the *SFXN4* gene was highlighted as the unique candidate gene of the QTL because it was approximately 1 Mb away from the leading SNP (WU_10.2_14_139488835) (Fig. 5A). Moreover, the expression level of *SFXN4* was significantly correlated with IMF (*r* = 0.28, FDR = 4.00*E*^−4^). Although no cis or trans-eQTL signal was identified for *SFXN4*, six SNPs located in its 3’UTR displayed significant allele-specific expression (Fig. 5C).

In addition, 117 SNPs were associated with IMF on the significant level of *p*-value ≤ 1.00*E*^−3^ (Fig. 5A & 5B), which represented 1.94% of all tested SNPs. By integrated analysis of GWAS, eQTL and IMF-gene correlation analysis, we finally highlighted 9 SNPs that satisfied the triple relationship of SNP-gene-trait, involved in three genes, namely *AGT*, *EMG1* and *PCTP* (Table 2). Taking the *AGT* gene as an example, its expression level was significantly correlated with IMF (*r* = 0.34, FDR = 1.00*E*^−5^), and the polymorphism of ALGA0078039 was associated with IMF (*p*-value = 1.06*E*^−4^) and the expression level of *AGT* (*p*-value = 1.02*E*^−3^).

**Table 2:** The overlap between eQTL analysis, GWAS analysis and correlation analysis

SNP_id	Gene_Name	Correlation analysis			eQTL analysis				GWAS analysis	
		cor	***p***value	FDR	r	***p***value	FDR	type	r	***p***value
ALGA0078039	AGT	0.34	6.09E-06	1.00E-05	0.26	1.02E-03	4.17E-02	cis	0.31	1.06E-04
WU_10.2_5_66693369	EMG1	0.21	5.76E-03	6.20E-03	0.69	7.32E-23	2.10E-18	cis	0.28	5.29E-04
M1GA0007834	EMG1	0.21	5.76E-03	6.20E-03	0.66	2.85E-20	3.58E-16	cis	0.27	7.90E-04
ALGA0032380	EMG1	0.21	5.76E-03	6.20E-03	0.52	7.12E-12	6.03E-09	cis	0.27	8.70E-04
ALGA0032795	EMG1	0.21	5.76E-03	6.20E-03	−0.52	7.46E-12	2.90E-06	trans	−0.27	7.06E-04
ASGA0026250	EMG1	0.21	5.76E-03	6.20E-03	−0.52	7.46E-12	2.90E-06	trans	−0.27	7.06E-04
WU_10.2_5_73855132	EMG1	0.21	5.76E-03	6.20E-03	−0.52	7.46E-12	2.90E-06	trans	−0.27	7.06E-04
WU_10.2_5_73348031	EMG1	0.21	5.76E-03	6.20E-03	−0.50	3.61E-11	1.14E-05	trans	−0.26	9.92E-04
ALGA0065989	PCTP	0.21	6.43E-03	7.30E-03	−0.35	6.72E-06	8.81E-04	cis	−0.30	1.58E-04

“cor” represents the Pearson correlation coefficient. “r” represents the correlation coefficient evaluated by matrixEQTL

## Discussion

In the present study, the IMF content of the F1 animals of Luchuan sows and Duroc boars displayed a great range of variation, from 0.77 to 7.36%, which was higher than that of Berkshire pigs (from 0.42 to 3.83%) [10]. Too much intrapopulation variation was a disadvantage for commercial production, and therefore, breeders were seeking genetic improvement of this trait. However, to our knowledge, the genetic basis of the IMF content of this population has not been well studied. Thus, identification of IMF-correlated genes could help guide the discovery of candidate genes and genetic markers and could benefit the research on genetic improvement.

By correlation analysis, we identified 212 IMF-correlated genes. Within the 212 IMF-correlated genes, *FASN*, *PLIN1*, *PIN1*, *CEBPA*, *CIDEC*, *SFRP5*, *SDR16C5*, and *SLC9A3R1* were identified to be DEGs in the muscle of Duroc pigs with extreme lipid profiles [9]. And *SCD*, *FASN, PLIN1*, *THRSP*, *CIDEC*, *ALDOC*, *AGT*, *PC*, and *CA5B* were identified to be DEGs in the muscle of Berkshire pigs with extreme IMF content [10]. In addition, *LEP*, *SCD*, *MT3*, *PENK*, *B9D2*, *CYP4B1*, *KIAA2013* and *ADAMTSL3* were also identified to be DEGs in the muscle of Iberian × Landrace back-crossed pigs with extreme IMF content [14]. Therefore, trait correlated expression analysis confirmed some of the DEGs related to IMF, which supported the reliability of our results. And compared with the strategy of comparative transcriptome study, trait correlated expression analysis depended on a large population that ensured relatively higher statistical power and reliability of results.

The comparative results mentioned above revealed some common functional genes, such as *SCD*, *PLIN1*, and *FASN*, all of which came from the AMPK/PPAR signaling pathway, indicating the commonality of the signaling pathway involved in the regulation of IMF deposition in different pig breeds. In agreement with the result of GO analysis, both KEGG and GSEA revealed that the IMF-correlated genes were mainly enriched in the processes of fatty acid and energy metabolism, both of which were regulated by the AMPK/PPAR signaling pathway [15]. As a kinase activated by AMP, AMPK plays a key role in the regulation of cellular and overall energy balance, and its activation accelerates ATP production by promoting fatty acid oxidation and glucose transport [15–18]. In addition, AMPK could affect lipolysis by regulating the activity of adipose triglyceride lipase (ATGL) and hormone-sensitive lipase (HSL) [15, 17], reduce fatty acid synthesis through phosphorylation and inactivation of acetyl CoA carboxylase (ACC) [15, 18], and inhibit fatty acid oxidation by inhibiting fatty acid translocase (CD36) that played a vital role in the homeostasis of lipid metabolism [15, 19, 20]. As a downstream pathway of AMPK, PPARs act as fatty acid sensors and play a crucial role in lipid metabolism, in which PPAR gamma (PPARγ) acts as a major activator of adipogenesis and fatty acid storage [21, 22]. Together, the AMPK/PPAR signaling pathway plays a vital role in regulating IMF content, which was further supported by in vitro [23, 24] and in vivo experiments [25, 26] as well as with transgenic models [27].

Trait correlated expression analysis highlights functional genes that may affect the phenotype by changing the expression levels, eQTL and ASE analyses provide direct clues for characterization of genes carrying genetic variant modulating its expression level, so integrated analysis of both strategies will be an efficient way to prioritize functional candidate genes for complex traits. In the present study, we highlighted 99 IMF-correlated genes that were the eQTL or ASE associated genes identified in our previous study [13]. Of these 99 genes, *SCD*, *ADIPOQ*, *FASN*, and *PCK1* were in the AMPK/PPAR signaling pathway, and the genetic polymorphisms of *SCD* [28–31], and *FASN* [32] were verified to be associated with the IMF content and fatty acid composition in pigs. However, so far, no causative mutation controlling IMF content has been identified from these genes, indicating further studies are necessary. Besides, the 99 genes contained some of novel functional candidate genes of IMF. For example, the *NAXE* gene encodes the NAD(P) HX epimerase, also known as ApoA-I binding protein (AIBP), that interacts with apolipoprotein A-I of high-density lipoproteins (HDL), which can regulate lipid rafts via cholesterol and HDL trafficking [33, 34]. And lipid raft microdomains can modulate the surface availability of CD36, which in turn affects fatty acid oxidation and uptake [15, 35, 36]. Another example is *PARK7*, also known as *DJ-1*, which regulates lipid raft-dependent endocytosis [37]. The deletion of *PARK7* could promote fatty acid oxidation and prevent hepatic steatosis in mice [38]. Therefore, the identification of strong cis-eQTL, ASE or trans-eQTL signals associated with those genes provided the directions for characterizing the causal variants regulating IMF content.

Trait correlated expression analysis together with eQTL or ASE analysis would benefit the characterization of functional candidate genes underlying the QTL identified by GWAS. By GWAS of IMF, we identified a QTL on chromosome 14. The *SFXN4* gene was regarded as the unique candidate gene for the QTL because it was approximately 1 Mb away from the leading SNP and its expression level was strongly correlated with IMF. In addition, *SFXN4* encodes a mitochondrial transmembrane protein that plays a key role in mitochondrial respiratory homeostasis [39], so we speculate that it may influence lipid metabolism by affecting energy metabolism, which still needs further investigation. Integration analysis of GWAS, eQTL analysis and trait-gene correlation analysis provided support for the candidacy of another three novel genes affecting IMF, namely *AGT*, *EMG1*, and *PCTP*. The *AGT* gene encodes angiotensinogen, a member of the process of lipid synthesis and metabolism, and its silencing attenuated lipid accumulation in adipocytes [40, 41]. *PCTP* encodes phosphatidylcholine transfer protein that has an important effect on lipid rafts and lipid transport by accelerating apolipoprotein A-I-mediated phospholipid and cholesterol efflux [42, 43]. *EMG1* encodes N1-specific pseudouridine methyltransferase involving in ribosome biogenesis [44], but little is known about its effect on lipid metabolism. Notably, the candidacy of these four novel genes affecting IMF needs to be investigated further.

In the present study, we did not find many SNP-gene-trait links. One obvious reason is the limited sample size, which was fine for eQTL, but small for GWAS. Another important reason is that the F1 animals is not suitable for GWAS due to its limited genetic segregation and power. In addition, the low resolution of SNPs in chips may result in the loss of a large amount of genetic information. Nevertheless, we believe the analysis approach establishing the triple relationship of SNP-gene-trait is interesting. Firstly, any relationship supports the rationality of the other two connections, which could justify the result. Secondly, the triple relationship characterized the causality relationship between gene (or quantitative trait gene, QTG) and phenotypic variation, and provide clues for identifying the quantitative trait nucleotide (QTN). To verify the advantage of this approach, a suitable population, a large sample size, and the use of high-density genotyping method such as sequencing are necessary for future study.

## Conclusions

Analysis of trait correlated expression revealed the vital role of the processes of lipid biosynthesis and energy metabolism and the AMPK/PPAR signaling pathway in regulating IMF content. Characterization of IMF-correlated genes that associated with eQTL or ASE signals will facilitate cloning causal genes of IMF. We further highlighted four candidate genes of IMF, but their effects on IMF need further study.

## Methods

### Animal sampling and phenotyping

The experimental population was described in our recent paper [13]. Briefly, it was composed of 425 F1 animals from the crossing of 8 Duroc boars and 158 Luchuan sows (DL pigs), which were fed with the same diets at the same farm. All animals were slaughtered by a standardized procedure at the age of 210 days ±6 days at the same abattoir. All animal procedures followed the guidelines of regulations for the administration of affairs concerning experimental animals, issued by the State Council of the People’s Republic of China. The current study was approved by the Scientific Ethics Committee of Huazhong Agricultural University (the approval number was HZAUSW-2016-010), Wuhan, China.

Twenty-five minutes postmortem, samples of the longissimus dorsi muscle at the thoracolumbar junction were taken for IMF content measurements and RNA extraction (stored in liquid nitrogen). IMF content was measured with the Soxhlet extraction method for each animal, with the following steps: 1) prepare ~ 60 g minced meat after removing the fascia and fat on the surface of the muscle; 2) dry the minced meat in a 100 °C oven, record the weight loss and calculate the content of water and dry matter; 3) package the powdered dried meat into three separate filter-paper bags for Soxhlet extraction with ether; and 4) calculate the fat content of each sample contained in a filter-paper bag and multiply it by the dry matter content, which yields the IMF content in the muscle. The final IMF content for one individual was the average value of three samples.

### RNA preparation and sequencing

The RNA preparation and RNAseq procedure were introduced in our recent paper [13]. Briefly, total RNA was extracted with Trizol reagent (Invitrogen, USA) from the *Longissimus dorsi* muscle of 189 DL pigs (a randomly selected subset of the DL population mentioned above). RNA concentration and integrity were accessed with NanoDrop 2000 spectrophotometers (Thermo Scientific, USA) and Agilent 2100 Bioanalyzer (Agilent Technologies, USA), respectively. The 150-bp, paired-end, nonstrand-specific libraries were prepared from one microgram of total RNA for each sample using NEBNext, Ultra RNA Library Prep kit for Illumina (NEB, USA) according to the manufacturer’s protocols. The cDNA yield of each library was quantified with Qubit (Invitrogen, USA), and the quality of the libraries was assessed using the Agilent 2100 Bioanalyzer (Agilent Technologies, USA). The prepared libraries were sequenced on the Illumina HiSeq 4000 platform in Shanghai Majorbio Bio-pharm Technology Co., Ltd. For each sample, at least 6.0 Gigabase pairs (Gb) of sequencing data were obtained. After the sequencing reaction was completed, clean reads were obtained from the raw reads through the following quality control steps: 1) removed adapter; 2) reads containing more than 5% N were removed; and 3) low-quality reads were removed, which were performed using SOAPnuke [45] software with the following parameters “-n 0.01 -l 20 -q 0.4 -A 0.25 --cutAdaptor -Q 2 -G --polyX 50 --minLen 150”. For the 189 animals, an average of 48.86 million reads was obtained, which was presented detailly in our recent paper [13].

### RNA-seq data analysis

The pipeline of RNA-seq data analysis was introduced in our previous study [13]. Briefly, the obtained RNA-seq data was aligned to the genome assembly Sscrofa11.1 (Ensembl Release 90) using HISAT2 V2.0.5 with a transcript annotation index [46]. The aligned reads for each sample were then assembled into genes with StringTie v1.3.3 [47], from which the expression level represented as TPM (transcripts per million mapped reads) value were estimated for each gene. Moreover, those genes whose TPM was greater than 0.01 in more than 90% of samples were used for correlation and eQTL analyses.

### Correlation analysis between IMF content and gene expression levels

The phenotypic data and gene expression levels were corrected using the following fixed linear model, Y = G + Ba + Bo + A + e, where Y represents the original IMF content or log_2_-transformed expression levels, G is gender, Ba is slaughter batch, Bo is the effect of boar, A represents age as a covariate, and e is a random residual. For all expressed genes, the Pearson correlation coefficients were calculated between the residuals of log_2_-transformed expression levels and IMF content using cor.test function in R. The statistical significance of correlation analysis was further evaluated by the false discovery rate (FDR) through 10,000 times random permutation, and the FDR by permutation smaller than 0.01 as the significance threshold of correlation. Of note, the most extreme FDR by permutation was set as 1.00*E*^−5^.

### Gene ontology and KEGG pathway analysis

Gene Ontology (GO) and Kyoto Encyclopedia of Genes and Genomes (KEGG) [48] analysis were carried out using the online software Metascape [49] (https://metascape.org/gp/index.html) with default parameter. In short, the default ontology sources including KEGG Pathway, GO Biological Processes, Reactome Gene Sets, Canonical Pathways and CORUM and so on, were used for gene enrichment analysis. Then, the statistical test based on cumulative hypergeometric distribution was carried out, and the Benjamini & Hochberg false-discovery rate approach was used for adjusting the *p*-value (q-value). Meanwhile, Metascape performed a hierarchical cluster analysis based on the similarity of terms. Furthermore, the significant level of the KEGG pathway and GO biological processes were filtered by setting a q-value less than 0.05.

### Gene set enrichment analysis (GSEA)

GSEA was performed by using the curated gene sets (C2, KEGG gene sets) of the Molecular Signature Database (MSigDB) v.5.2 [50]. All the genes ranked by their corresponding Pearson correlation coefficients were used for GSEA with default parameters following the standard procedure (http://www.broadinstitute.org/gsea/doc/GSEAUserGuideFrame.html). The GSEA software estimated the probability of the normalized enrichment score (NES) for each gene set, and adjusted the *p* value by FDR. We set the default FDR value (FDR < 0.25) as the level of statistical significance for GSEA.

### PPI network generation

The protein-protein interaction (PPI) network was constructed by STRING (https://string-db.org/) [51] using the multiple proteins process and choosing the “*Homo sapiens*” as background. To improve accuracy and intuitiveness, we set the minimum interaction score as 0.4, the number of k-means clusters as 8, and hid disconnected nodes. In addition, the core genes were evaluated by the degree of connectivity, and a sub-core network was built with the same condition.

### Overlapping analysis of IMF-correlated genes and eQTL/ASE genes

The characterized IMF-correlated genes were overlapped with the eQTL and ASE associated genes that were identified previously [13]. The pipeline of eQTL and ASE analysis was introduced in our previous study [13]. Briefly, the eQTL analysis was conducted with MatrixEQTL [52] using a fixed linear model, with sex, slaughter batch and boar as fixed effects, and age and top five principal components based on marker genotypes as covariates accounting for systematic variation. ASE analysis was carried out using the GATK ASEReadCounter tool [53] with the N-masking genome.

### GWAS of IMF

As described in our recent paper, the 189 animals were genotyped using the Illumina porcine 50 K + SNP iSelect™ BeadChip and 36,045 SNPs were kept after the step of quality control [13]. We noted that the mixed linear models fitting random effects of relationship matrix could lead to over-fitting of the model. Therefore, GWAS of IMF was performed using MatrixEQTL [52] with the same model as that used for the eQTL analysis [13], which was based on a linear regression analysis without fitting random effects. Unlike eQTL, the dependent variable was normalized IMF instead of normalized gene expression level. To highlight the IMF-associated SNPs, we set two different significance levels at *p* = 1.00*E*^−3^ and *p* = 1.39*E*^−6^, respectively.

## Supplementary Information

**Additional file 1: Supplementary Table S1.** The result of correlation analysis between gene expression level and IMF content

**Additional file 2: Supplementary Table S2.** Enrichment analysis result of 212 IMF-correlated genes with Metascape online tool

**Additional file 3: Supplementary Table S3.** The result of Gene set enrichment analysis (GSEA) analysis for all the 13, 450 expressed genes

## Data Availability

The RNAseq data used in the current study are available in the GEO database of NCBI with accession ID GSE124315 (https://www.ncbi.nlm.nih.gov/geo/query/acc.cgi?acc=GSE124315). The datasets supporting the conclusions of this article are included in the article and its Additional files.

## Abbreviations

ACC: acetyl CoA carboxylase
ASE: allele-specific expression
ATGL: adipose triglyceride lipase
CD36: fatty acid translocase
DEGs: differential expressed genes
eQTL: expression quantitative trait locus
QTG: quantitative trait gene
QTL: quantitative trait locus
QTN: quantitative trait nucleotide
FDR: false discovery rate
GO: Gene Ontology
GSEA: Gene Set Enrichment Analysis
GWAS: genome-wide association study
HDL: high-density lipoproteins
HSL: hormone-sensitive lipase
IMF: Intramuscular fat
KEGG: Kyoto Encyclopedia of Genes and Genomes
q-value: adjusting *p*-value
PPI: protein-protein interaction
RNA-seq: RNA sequencing
TPM: transcripts per million mapped reads
